# Exploring Patient Awareness and Perceptions of the Appropriate Use of Antibiotics: A Mixed-Methods Study

**DOI:** 10.3390/antibiotics6040023

**Published:** 2017-10-31

**Authors:** Marion E. Davis, Tsai-Ling Liu, Yhenneko J. Taylor, Lisa Davidson, Monica Schmid, Traci Yates, Janice Scotton, Melanie D. Spencer

**Affiliations:** 1 Center for Outcomes Research and Evaluation, Carolinas HealthCare System, Charlotte, NC 28203, USA; Tsai-Ling.Liu@carolinashealthcare.org (T.-L.L.); Yhenneko.Taylor@carolinashealthcare.org (Y.J.T.); Monica.Schmidt@carolinashealthcare.org (M.S.); Traci.Yates@carolinashealthcare.org (T.Y.); Melanie.Spencer@carolinashealthcare.org (M.D.S.); 2 Department of Infectious Disease, Carolinas HealthCare System, Charlotte, NC 28203, USA; Lisa.Davidson@carolinashealthcare.org; 3 Patient Experience, Carolinas HealthCare System, Charlotte, NC 28202, USA; Janice.Scotton@carolinashealthcare.org

**Keywords:** antibiotics, antibiotic resistance, beliefs about antibiotics, expectations about antibiotics, patient and provider communication, mixed-methods research

## Abstract

In the outpatient setting, estimates suggest that 30% of the antibiotics prescribed are unnecessary. This study explores patient knowledge and awareness of appropriate use of antibiotics and expectations regarding how antibiotics are used for their treatment in outpatient settings. A survey was administered to a convenience sample of patients, parents, and caregivers (n = 190) at seven primary care clinics and two urgent care locations. Fisher’s exact tests compared results by patient characteristics. Although 89% of patients correctly believed that antibiotics work well for treating infections from bacteria, 53% incorrectly believed that antibiotics work well for treating viral infections. Patients who incorrectly believed that antibiotics work well for treating viral infections were more than twice as likely to expect a provider to give them an antibiotic when they have a cough or common cold. Patients who completed the survey also participated in semi-structured interviews (n = 4), which were analyzed using thematic analysis. Patients reported experiencing confusion about which illnesses may be treated by antibiotics and unclear communication from clinicians about the appropriate use of antibiotics. Development of easy to understand patient educational materials can help address patients’ incorrect perceptions of appropriate antibiotic use and facilitate patient-provider communication.

## 1. Introduction

In the United States, antibiotic-resistant infections affect more than 2 million people each year, account for at least 23,000 deaths, and have a total economic burden that exceeds $20 billion in direct healthcare costs alone [1]. Overtreatment with antibiotics has been identified as the major factor leading to antibiotic resistance worldwide [1,2,3,4]. Approximately 30% of antibiotic prescriptions in US outpatient settings are considered to be unnecessary [5]. Therefore, efforts aimed at understanding patient and provider level factors that contribute to the over-prescribing of antibiotics in the outpatient setting are essential [6,7].

Evidence suggests that patient beliefs as well as their expectations and demands play a key role in inappropriate antibiotic prescribing by healthcare providers in outpatient settings [8]. Patients who expect to receive antibiotics at an outpatient visit were prescribed antibiotics more frequently than those who were not expecting them [9,10,11,12,13]. Likewise, providers report feeling pressured to prescribe antibiotics even when they believe that antibiotics are not clinically indicated [12,14,15,16,17]. Studies conducted among U.S. and international samples of patients have identified age, education, and socioeconomic status as factors associated with patient knowledge of and expectations regarding the use of antibiotics [18,19]. Research conducted among patients in U.S. primary care settings that uncovers patient beliefs about antibiotics and how they are related to patient expectations regarding the use of antibiotics in their treatment can expand our understanding of the factors associated with overprescribing and guide interventions aimed at decreasing overuse and inappropriate use of antibiotics.

The aims of this study were to explore beliefs about antibiotics and knowledge and awareness of the appropriate use of antibiotics and antibiotic resistance among patients in primary care settings and to examine how their beliefs, knowledge, and awareness may be related to their expectations regarding the use of antibiotics in their treatment. We used a mixed-methods approach that included the use of a survey and semi-structured interviews to understand both the prevalence of misconceptions about antibiotics and to provide a more detailed context for patient beliefs about antibiotic use. Although this approach has been used in prior studies to examine patient perceptions of the use of antibiotics in their treatment in outpatient settings [8], this study adds to the literature through its focus on the identification of beliefs and other factors that impact patient expectations regarding the use of antibiotics in their treatment and how to improve patient-provider communication about the appropriate use of antibiotics in outpatient settings in the U.S.

## 2. Materials and Methods

### 2.1. Study Design and Setting

This study was conducted at primary care clinics and urgent care locations that are part of a large integrated health care system in North Carolina with a network of 40 hospitals, over 180 physician offices, academic medical centers, and long term care facilities. We used a sequential explanatory mixed methods approach whereby we conducted a survey, analyzed the results from the survey, and used the survey findings to inform the development of questions for semi-structured interviews. A multi-disciplinary team that included researchers, physicians, infectious disease specialists, pharmacists, quality improvement coaches, and a health literacy expert implemented the project. Approval to conduct this study was obtained from the Institutional Review Board of the health care system where the study was conducted.

### 2.2. Survey Method

After completing a literature review, an iterative process was used to develop a 16-item paper-based survey that included validated measures as well as newly developed measures of concepts of interest. To measure patient knowledge of the appropriate use of antibiotics, we asked respondents to indicate how well antibiotics work for treatment of infections from bacteria such as strep throat or some sinus infections and for treatment of infections from a virus such as the flu or common cold (using a Likert response scale where 1 = Not well at all and 5 = Extremely well and that included a do not know option was included as well). To measure patient awareness of antibiotic resistance, we asked respondents to indicate how much they have heard about the antibiotic resistance problem (using a response scale that included a great deal, a fair amount, some information, nothing and also a do not know option). To measure their perceptions of the outcomes of antibiotic resistance, we asked respondents to indicate what they think will happen when a person takes an antibiotic that is not needed to treat their illness (using a yes/no response scale that also included a do not know option). To measure their expectations regarding the prescribing of antibiotics by providers, we asked respondents to indicate what they expect their provider to give them when they have a cough or a common cold (using a yes/no response scale that also included a do not know option). To measure patient preferences for treatment, we asked respondents to indicate what their provider could do to make them feel better when an antibiotic was not prescribed to treat their illness (using a yes/no response scale that also included a do not know response option). It also recorded demographic characteristics including gender, age, level of education, and ethnicity. The survey had a reading level accessible for most patients and could be self-administered (Supplementary Materials).

The paper-based survey was administered to a convenience sample of 200 adult patients aged 18 or older at seven (7) primary care clinics and two (2) urgent care locations. Participants were recruited between September and October of 2016 using flyers posted in the primary care clinics and urgent care locations and through onsite recruitment in the clinic waiting areas by trained quality improvement coaches. Survey instructions informed participants of the research purpose of the survey and that their participation was voluntary. Participants received a water bottle for their participation. Survey responses with non-missing data on age, gender, and education (n = 190) were included in the final analyses.

### 2.3. Survey Data Analysis

The data from the survey were input into the REDCap electronic data capture tools (REDCap, Nashville, TN, USA) and managed in REDCap as well. Survey data were summarized using means and percentages as appropriate and Fisher’s exact tests were used to compare differences between groups categorized by gender, level of education, and parental status. Multivariate logistic regression models examined associations between patient expectations regarding the use of antibiotics in treatment and patient knowledge level of the appropriate use of antibiotics. The models were adjusted for age, gender, and education, and the results reported as adjusted odds ratios (AOR). All tests were two-sided and *p*-value < 0.05 indicated significance. Statistical analyses were conducted using SAS version 9.4 (SAS Institute, Cary, NC, USA). 

### 2.4. Qualitative Research Method

Among the 200 patients who completed the survey, 64 agreed to be contacted by phone, e-mail or mail to learn more about the qualitative research. Among the 64 individuals who were contacted, only 4 individuals agreed to participate in a qualitative, semi-structured interview either in person or via telephone. A research team member used a prepared script to schedule the semi-structured interviews. All of the participants in the interviews were female, had college level education, and were between 25 and 45 years old. The interviews were conducted via telephone or in-person, were thirty minutes in length, and took place between November and December of 2016. Participants received a $25 gift card.

The interview guide explored in-depth the same concepts examined in the survey including: beliefs about antibiotics, knowledge of the appropriate use of antibiotics, awareness of antibiotic resistance, expectations regarding the use of antibiotics in their treatment, and preferences regarding what providers could do to make them feel better if they were not given a prescription for an antibiotic. The interviews were audio recorded, transcribed, and analyzed using the following process: (1) each interview transcript was analyzed to identify key themes; (2) a code book was developed based on the key themes identified in the transcripts using NVivo version 10 (QSR International, Burlington, MA, USA); (3) the code book was used to code every statement in each transcript; and (4) pattern coding analysis was used to organize major themes under concepts and to explain them.

## 3. Results

### 3.1. Survey Results

Table 1 displays the demographic characteristics of the survey respondents. The average age was 46 years. Respondents were largely female (77%), non-Hispanic White (71%), college educated (71%), and were not parents of children who had taken an antibiotic in the past 2 years (75%).

#### 3.1.1. Knowledge and Awareness of Appropriate Use of Antibiotics

Results regarding knowledge and awareness of appropriate antibiotic use are presented in Table 2. Most survey respondents (89%) correctly believed that antibiotics work extremely/very/somewhat well for treating infections from bacteria. However, patients with college level education were significantly more likely than those without college level education to correctly believe that antibiotics work extremely/very/somewhat well for treating infections from bacteria (93% vs. 80%, *p* = 0.02). More than half of survey respondents (53%) incorrectly believed that antibiotics work extremely/very/somewhat well for treating infections from a virus such as the flu or common cold. This incorrect belief did not differ significantly by gender, parental status, or education level.

#### 3.1.2. Awareness of Antibiotic Resistance and Knowledge of Outcomes of Antibiotic Resistance

Nearly two-thirds (62%) of the respondents reported that they have heard a great deal/fair amount about antibiotic resistance. Those with college level education were significantly more likely than those without college level education (72% vs. 55%, *p* = 0.03) to correctly believe that if someone takes an antibiotic unnecessarily, it may not work well in treating that person’s illness the next time. Similarly, those with college level education were significantly more likely than those without college level education (58% vs. 41%, *p* = 0.04) to believe that taking an antibiotic unnecessarily can weaken how well the antibiotic will work for other people in the future (Table 2).

#### 3.1.3. Expectations Regarding Treatment for Cough or Common Cold

Respondents were asked to indicate what they expect their provider to give them when they have a cough or common cold. When selecting from among the list of possible responses, most respondents (86%) reported that they expect their provider to give them tips on how to help them feel better. Eighty-five percent reported that they expect to receive information to assure them that their illness is not something worse. Nearly three-fourths (72%) reported that they expect to receive a prescription for something to help their symptoms and nearly one-fourth (22%) of respondents indicated that they expect their provider to give them a prescription for an antibiotic. Adjusted logistic regression analysis revealed that those who believed that antibiotics work extremely/very/somewhat well for treating infections from a virus such as the flu also had 2.76 times higher odds of expecting the provider to give them antibiotics when they have a cough or a common cold compared to those who did not believe that antibiotics worked well for a virus (Table 3).

#### 3.1.4. Preferences for Alternatives to Receiving an Antibiotic

Respondents were asked to indicate what their provider could do to make them feel better if their provider did not give them a prescription for an antibiotic. When selecting from among the list of possible responses, most respondents (78%) indicated that they want their provider to suggest an over-the-counter medicine that could help their symptoms. Patients with college level education were significantly more likely than those without college level education to say they would want their provider to suggest an over-the-counter medicine that could help their symptoms, call them within 24 to 48 hours to see if they feel better, and give them coupons for an over-the-counter medicine that could help their symptoms (Table 4).

### 3.2. Qualitative Results

**Figure 1:**
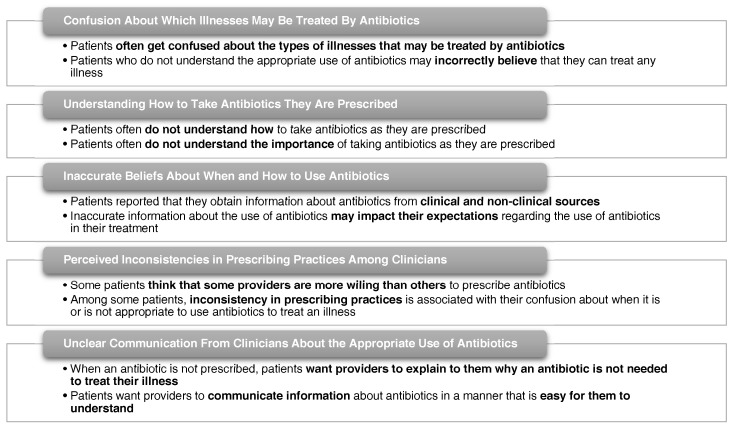
Themes emerging from semi-structured interviews with patients.

A thematic analysis of the semi-structured interview data identified the following barriers that patients face to improving their understanding of the appropriate use of antibiotics: confusion about which types of illnesses may be treated by antibiotics; understanding how to take antibiotics as they are prescribed; beliefs about when and how to take antibiotics that are informed by inaccurate information; inconsistent prescribing practices among clinicians; and unclear communication from clinicians about the appropriate use of antibiotics (Figure 1).

#### 3.2.1. Confusion about Which Illnesses May Be Treated by Antibiotics

Some patients were confused about the types of illnesses for which antibiotics are appropriate. When attempting to explain their knowledge of appropriate antibiotic use, one patient said: 

“… I don’t think they work for those [bacterial infections].… I had that backwards… The viral is the one that I don’t think it’s effective for. The bacterial [infections is what] I think that it does work for. I had that backwards...”

Patients who do not understand the appropriate use of antibiotics also may incorrectly believe that antibiotics can be used to treat any illness. 

“The average person probably thinks that whenever you feel bad, you can have an antibiotic. I don’t know a lot but I do know that it depends on what’s wrong with you… whether it will help you or not. But because they usually work so fast, people just [say], ‘Oh just give me an antibiotic.’”

“…they want to use it for everything and … it doesn’t help for everything…”

#### 3.2.2. Understanding How to Take Antibiotics as They Are Prescribed

Patients often do not understand how to take antibiotics as they are prescribed or the importance of taking antibiotics as they are prescribed. One patient explained: 

“It does start working really fast. So, if they get it [for] 14 days, and they start feeling better by day two, you know some people will stop taking it, which cuts down on its effectiveness… if you don’t take it for however long they say take it.”

#### 3.2.3. Inaccurate Beliefs about When and How to Use Antibiotics

Patients indicated that they obtain information about antibiotics from clinical and non-clinical sources, such as their provider, family members, friends, and websites. These sources may inform their beliefs about when and how to use antibiotics and may impact their beliefs about if they need a provider to tell them when an antibiotic is needed to treat their illness. They explained:

“There are so many people that are not medically educated… I think that’s something that we should leave up to the doctor… because we didn’t go to school for that... I think a lot of people don’t know when they really need it.”

“… It’s not a pill that covers everything. There [are] some things that antibiotics don’t fix… You would need your doctor or nurse practitioner to tell you that you have strep or [that] you have whatever… I don’t think you should be able to… diagnose yourself and [say], ‘I need an antibiotic because I [have] a cough.’”

#### 3.2.4. Perceived Inconsistencies in Prescribing Practices Among Clinicians

Among some patients, perceptions that some clinicians are more willing than others to prescribe antibiotics were associated with their confusion about when it is appropriate to treat an illness with an antibiotic. They explained:

“I don’t really have a doctor that [I have] been seeing…that has that type of relationship with me. So, they may be more hesitant to give me something [because] they don’t know me on a personal level. I think the personalization… plays a part in prescriptions.” 

“I know this sounds bad, but if you go to urgent care, they’ll give you anything.”

#### 3.2.5. Unclear Information from Clinicians about Appropriate Use of Antibiotics

When a provider does not prescribe an antibiotic, patients want them to clearly explain why they do not need an antibiotic to treat their illness. Patients suggested that this will build trust with their providers. 

“Tell us why so that we [can] do a better job with taking it the right way or tell us why so that we don’t ask for it every single time we come to the doctor…so we [can] understand [that] it doesn’t fix everything. The average person doesn’t understand that it doesn’t fix everything. So maybe if we could get some education on that.”

“The more I know, the better I understand why something is the way it is…”

Patient responses indicated that they want providers to communicate about antibiotics in a manner that is easy for them to understand.

“I think [doctors] could do a little better job of educating… the patient or individual on … the do’s and don’ts...”

“I have a good provider who educates you… I’ve been to doctors before where they just don’t explain anything. They told you that you were sick or whatever and they wrote you a prescription and sent you out the door… [without] explaining what the prescription was or what it was for…[or] how effective it could be… [in] dealing with your situation.”

## 4. Discussion

This study explored patient beliefs about antibiotics, their knowledge and awareness of the appropriate use of antibiotics and antibiotic resistance, and their expectations regarding the use of antibiotics in their treatment. Our findings are consistent with prior research. In a large survey of U.S. consumers, 26% reported expecting an antibiotic from their provider during a visit for a cough or cold [14]. In our sample, 22% reported having a similar expectation. Also, surveys with international samples found that individuals with lower levels of education were less likely to understand the appropriate use of antibiotics compared to those with higher levels of education [20,21]. Our study also found that those with a lower level of education were less likely to correctly understand the appropriate use of antibiotics than those with a higher level of education. We also found that those with college level education differ from those with a lower level of education on their preferences for what a provider could do to help them feel better if they are not given an order for antibiotic. More research should be conducted to better understand factors that may impact patient preferences for how to make them feel better.

Our study adds to existing knowledge by highlighting when and how to communicate with patients about the appropriate use of antibiotics in their treatment. Results from the survey and semi-structured interviews revealed that patient beliefs regarding antibiotics are often informed by inaccurate information that impacts their expectations regarding the use of antibiotics in their treatment. Semi-structured interviews also revealed that patients think that providers should communicate in a manner that is easy for patients to understand and clearly explain why an antibiotic is not needed to treat an illness. These findings suggest that sharing information with patients about the appropriate use of antibiotics prior to a visit with a provider may help patients to develop more realistic expectations regarding the use of antibiotics in their treatment and that the use of health literate educational materials may help improve communication between providers and patients about the appropriate use of antibiotics.

### Strengths and Limitations of Study

One strength of our study is the use of a sequential mixed-methods approach to examine patient knowledge of the appropriate use of antibiotics, awareness of antibiotic resistance and their expectations regarding the use of antibiotics in their treatment. We surveyed 200 patients and completed semi-structured interviews with 4 patients who completed the survey and also agreed to participate in the qualitative research. We used the findings from the survey to develop questions that were explored in the semi-structured interviews. The triangulation of research methods enabled us to corroborate consistencies in the findings observed from each methodology. Both methodologies revealed that patients often do not understand which illnesses may be treated by antibiotics and that patient expectations regarding the use of antibiotics in their treatment are informed by their level of understanding of the appropriate use of antibiotics. The findings from the semi-structured interviews also provided insights into the types of barriers that patients face when seeking to obtain information about the appropriate use of antibiotics and insights regarding how to design patient education materials that will be easy for them to understand and that may be used by providers when they are discussing antibiotics with patients. This study had limitations. The survey and the semi-structured interviews were conducted among a convenience sample of patients. Hence, the results may not be representative of the entire patient population. However, the findings from the current survey and semi-structured interviews were consistent with findings from prior research studies conducted with nationally representative random samples in the US and international samples that revealed that individuals often incorrectly believe that taking an antibiotic will make them feel better when they have a common cold. Similar to our study, previous work has highlighted that patients were not aware of the dangers associated with taking antibiotics when they are not needed to treat their illness [14,19]. The semi-structured interviews were conducted among a small sample of individuals who agreed to participate in the qualitative interviews. However, the demographic profile of the individuals who participated in the qualitative interviews (e.g., all were female, all had some college education, and all were between the ages of 25 and 45) is similar to the demographic profile of those who participated in the survey (e.g., 75% were female, 71% had college level education, and 61% were between the ages of 25 and 45). Also, the thematic analysis of the qualitative data revealed findings that were consistent with findings from the survey research and therefore provide support that qualitative data saturation was achieved [22]. We had a low level of male participation in the survey. However, a review of the literature did not reveal a consistent finding among studies that compared males and females on knowledge of the appropriate use of antibiotics and behaviors associated with appropriate antibiotic use. Therefore, there is a need for additional research to be conducted on this topic [19,23,24,25]. Also, we used non-validated survey items to measure concepts if the review of the literature revealed that there were no existing validated measures to use for them. Finally, the survey sample was predominantly comprised of those with college level education.

## 5. Conclusions

Reducing the inappropriate use of antibiotics in outpatient settings will require the development of strategies that address both patient and provider level factors that impact prescribing practices. This study identified a specific need for communication support materials that help providers to clearly explain to patients when an antibiotic is or is not needed to treat their illness. This strategy component, along with appropriate patient level education materials that are easy to understand, is essential to help patients set realistic expectations regarding the role of antibiotics in their treatment.

## Figures and Tables

**Table 1:** Characteristics of the respondents (n = 190).

Characteristics	n	%
All	190	
**Age group**		
24 or under	16	8.4
25–34	43	22.6
35–44	44	23.2
45–54	31	16.3
55–64	25	13.2
65 or above	31	16.3
**Gender**		
Male	43	22.6
Female	147	77.4
**Parent of child taking antibiotic in past 2 years**		
Yes	47	24.7
No	143	75.3
**Education**		
College Education	134	70.5
No College Education	56	29.5
**Race**		
Non-Hispanic White	135	71.1
Non-Hispanic Black	31	16.3
Hispanic	12	6.3
Others	12	6.3

**Table 2:** Knowledge of antibiotics and antibiotic resistance among primary care patients (n = 190).

Characteristics	Antibiotics Work Extremely/Very/Somewhat Well For				When Someone Uses antibiotics When It Is Not Needed			
	Infections from Bacteria Such as Strep Throat Or Some Sinus Infections		Infections from a Virus Such as the Flu or the Common Cold		The Antibiotic Will Not Work Well in Treating that Person’s Illness the Next Time		It Can Weaken How Well the Antibiotic Will Work FOR Other People in the Future	
	n (%)	*p*-Value *	n (%)	*p*-Value *	n (%)	*p*-Value *	n (%)	*p*-Value *
All	169 (88.9)		101 (53.2)		128 (67.4)		101 (53.2)	
**Gender**		0.580		0.491		0.466		0.224
Male	37 (86.0)		25 (58.1)		27 (62.8)		19 (44.2)	
Female	132 (89.8)		76 (51.7)		101 (68.7)		82 (55.8)	
**Parent of child taking antibiotic in past 2 years**		0.604		0.740		1.000		0.866
Yes	43 (91.5)		26 (55.3)		32 (68.1)		24 (51.1)	
No	126 (88.1)		75 (52.4)		96 (67.1)		77 (53.8)	
**Education**		0.021		0.526		0.028		0.038
College Education	124 (92.5)		69 (51.5)		97 (72.4)		78 (58.2)	
No College Education	45 (80.4)		32 (57.1)		31 (55.4)		23 (41.1)	

* *p*-value comparing knowledge by patient characteristics from Fisher’s Exact test.

**Table 3:** Association between patient expectations for an antibiotic when they have a cough or common cold and knowledge of the appropriate use of antibiotics.

Characteristics	When You Have a Cough or a Common Cold, You Would Expect Your Provider to Give You Antibiotic		
	OR	95% CI	*p*-Value
**Knowledge of appropriate use of antibiotics**			
Yes	-	-	-
No	2.76	(1.67–4.57)	<0.001
**Age (per 1 point increase)**	0.97	(0.95–1.00)	0.030
**Gender**			
Male	-	-	-
Female	0.75	(0.49–1.16)	0.199
**Education**			
College Education	-	-	-
No College Education	1.43	(0.96–2.13)	0.079

**Table 4:** Patient preferences for what their provider could do to make them feel better if they do not give them an antibiotic (n = 190).

Characteristics	If They Do Not Give You an Antibiotic, What Could Your Provider Do to Make You Feel Better?									
	Suggest an Over-The-Counter Medicine That Could Help My Symptoms		Give Me an Order for a Medicine That Could Help My Symptoms		Tell Me Where I Could Learn more about My Illness (such as a Website or Handout)		Call me in 24 to 48 h to See If I Feel Better		Give Me Coupons for an Over-The-Counter Medicine That Could Help My Symptoms	
	n (%)	*p* *	n (%)	*p* *	n (%)	*p* *	n (%)	*p* *	n (%)	*p* *
All	149 (78.4)		115 (60.5)		69 (36.3)		57 (30.0)		69 (36.3)	
**Gender**		0.140		1.000		0.107		0.185		0.212
Male	30 (69.8)		26 (60.5)		11 (25.6)		9 (20.9)		12 (27.9)	
Female	119 (81.0)		89 (60.5)		58 (39.5)		48 (32.7)		57 (38.8)	
**Parent of child taking antibiotic in past 2 years**		0.689		0.126		1.000		1.000		0.301
Yes	38 (80.9)		33 (70.2)		17 (36.2)		14 (29.8)		14 (29.8)	
No	111 (77.6)		82 (57.3)		52 (36.4)		43 (30.1)		55 (38.5)	
**Education**		0.004		1.000		0.186		0.023		0.020
College Education	113 (84.3)		81 (60.4)		53 (39.6)		47 (35.1)		56 (41.8)	
No College Education	36 (64.3)		34 (60.7)		16 (28.6)		10 (17.9)		13 (23.2)	

* *p*-value comparing preferences by patient characteristics from Fisher’s Exact test.

## Supplementary Materials

The following are available online at www.mdpi.com/2079-6382/6/4/23/s1. A complete copy of the survey that was administered to study participants has been provided.
