# Supplementary material for: Exploring Patient Awareness and Perceptions of the Appropriate Use of Antibiotics: A Mixed-Methods Study
Source: Antibiotics (Basel). 2017 Oct 31;6(4):23. doi: 10.3390/antibiotics6040023 (PMC5745466; doi:10.3390/antibiotics6040023)
Supplement: Supplementary file 1 [file antibiotics-06-00023-s001.pdf]

antibiotics

MDPI

Article

Exploring patient awareness and perceptions of the appropriate use of antibiotics: a mixed-methods study

Marion E. Davis [1,*], Tsai-Ling Liu [1], Yhenneko J. Taylor [1], Lisa Davidson [2], Monica Schmid [1], Traci Yates [1], Janice Scotton [3], and Melanie D. Spencer [1]

Description

1. Please tell us about yourself (Select All That Apply)
   - I am the patient. I have taken an antibiotic within the past 2 years
   - I am the parent of a child who has taken an antibiotic within the past 2 years
   - I am caregiver to a person who has taken an antibiotic within the past 2 years
   - I am the patient, parent of a child, or caregiver to a person who has taken an antibiotic more than 2 years ago
   - I have never taken an antibiotic
   - I don't know

2. How long has it been since you were given an order for an antibiotic?
   - Less than 6 months
   - 6 to 12 months
   - More than 1 year
   - I have never gotten an order for an antibiotic
   - I don't know

Knowledge of Appropriate Use of Antibiotics

3. How well do antibiotics work for treating these kinds of infections?

	Not well at all 1	Not too well 2	Somewhat well 3	Very well 4	Extremely well 5	I don't know
Infections from bacteria such as strep throat or some sinus infections						
Infections from a virus such as the flu or common cold						

Experiences with Use of Antibiotics

4. When an antibiotic was ordered, were you taught how to take it by a provider?
   - Yes, I was taught how to take it (such as take it with food or take it for 7 days)
   - No
   - I don't know
   - I have never been given an order for an antibiotic
5. Have any of these been a problem for you?

	**Yes**	**No**	**I don't know**
The cost of an antibiotic			
Finding a provider who will write an order for an antibiotic			
Taking time away from work, school, caregiving or other matters to go see a provider			

Expectations of Providers Regarding Antibiotics

6. Do you think that some providers are more willing than others to write an order for an antibiotic?
    - Yes
    - No
    - I don't know
7. When you have a cough or a common cold, do you expect your provider to give you any of these?

	**Yes**	**No**	**I don't know**
Tips on how to help me feel better			
An antibiotic			
An order for something to help my symptoms			
Information to assure me that it is not something worse			

8. You ask your provider for an antibiotic. Your provider tells you that an antibiotic is not needed for your illness. What would you do? Please select all that apply.
    - Do what my provider says even if I don't get an antibiotic
    - Ask a provider in the same office to give me an antibiotic
    - Go to another Carolinas HealthCare System place/office to try to get an antibiotic
    - Go to a non-Carolinas HealthCare System place/office to try to get an antibiotic
    - Search for the antibiotic online and order it
    - File a complaint

9. If they do not give you an antibiotic, what could your provider do to make you feel better? Please select all that apply.
    - Suggest an over-the-counter medicine that could help my symptoms
    - Give me an order for a medicine that could help my symptoms
    - Tell me where I could learn more about my illness (such as a website or handout)
    - Call me in 24 to 48 hours to see if I feel better
    - Give me coupons for an over-the-counter medicine that could help my symptoms
    - Other: (Please tell us what would help you to feel better)

Level of Awareness of Antibiotic Resistance

10. Some antibiotics no longer work to fight infections. This problem is called antibiotic resistance or a superbug. How much have you heard about this problem?
    - Great deal
    - Fair amount
    - Some information
    - Nothing
    - I don't know

Perceptions of Outcomes of Antibiotic Resistance

11. Do you think any of these will happen when a person takes an antibiotic that is not needed to treat their illness?

	**Yes**	**No**	**I don't know**
The antibiotic will not work well in treating that person's illness the next time			
It can weaken how well the antibiotic will work for other people in the future			

Demographics

12. Are you Male or Female?
    - Male
    - Female
    - Prefer not to answer

13. In what year were you born? _____________
14. What is the last year or grade of school that you finished?
    - Grade school
    - High School but did not Finish
    - High School Diploma or GED
    - Business, Technical or Vocational School
    - Some College
    - Two-Year or Associates College Degree
    - Four-Year College Degree
    - Graduate Degree
15. Are you Spanish, Hispanic, or Latino / Latina origin or descent?
    - Yes
    - No
16. What is your race? Please choose one or more
    - White
    - Black or African American
    - Asian
    - Native Hawaiian or Other Pacific Islander
    - American Indian or Alaska Native
    - Prefer not to answer
    - Other (Please write your race below)

© 2017 by the authors. Submitted for possible open access publication under the terms and conditions of the Creative Commons Attribution (CC-BY) license (http://creativecommons.org/licenses/by/4.0/).
